# Gamification for the Win in Internal Medicine Residency: A Longitudinal, Innovative, Team-Based, Gamified Approach to Internal Medicine Board-Review

**DOI:** 10.7759/cureus.22822

**Published:** 2022-03-03

**Authors:** Allie H Dakroub, Jarrett J Weinberger, Diane L Levine

**Affiliations:** 1 Internal Medicine-Pediatrics, University of Pittsburgh Medical Center, Pittsburgh, USA; 2 Internal Medicine, Detroit Medical Center, Wayne State University, Detroit, USA

**Keywords:** longitudinal, board review, game-based learning, team based learning, gamification

## Abstract

Background: Game-based learning is an engaging and effective educational strategy in medical education. The Internal Medicine resident board review at our institution was considered dull and poorly attended by trainees. We hypothesized that a gamified, longitudinal, team-based approach to board review would rejuvenate board review and improve learner perception of quality and attendance.

Methods: We sought to improve the resident perception of and participation in board review through an innovative longitudinal, team-based, game-based intervention, the “Cohort Cup”. The “Cohort Cup” was developed and implemented over a 22-week intervention period from November 2017 to May 2018. Teams (cohorts) competed in real-time against one another. Evaluation methods include a pre/post attitudes survey on a 5-point Likert scale (1 - strongly disagree, 5 - strongly agree) and attendance data.

Findings: Of 105 residents eligible to participate, 82 completed the pre-intervention survey, and 74 completed the post-intervention survey. We observed statistically significant increases in self-perceptions of engagement, the perceived value of the sessions, and preferences for game-based learning. Self-perceptions of learner engagement improved from 2.74 to 3.8. The value of the educational experience increased from 2.68 to 3.95. Preferences for game-based learning improved from 3.77 to 4.32. Board review attendance doubled. Residents commented the intervention improved class bonding. Board passage rate increased from 86% to 97%.

Conclusions: Our game-based intervention successfully rejuvenated our board review. We observed more joy in the learning environment and improvements in resident engagement, and in their attitudes regarding board review. Game-based learning can be a valuable educational tool and can be a positive facet of educational communities.

## Introduction

Board review at our urban, academic Internal Medicine residency program was poorly attended with poor resident perceptions of utility and effectiveness. There was a general malaise regarding board review that negatively affected attendance, attitudes, participation, and, consequently, its perceived effectiveness. Many sessions were somewhat passive and unengaging. Residents did not view the board review as a coherent educational activity. Measurement of resident success was only assessed by passage on the American Board of Internal Medicine (ABIM). Most fundamentally, board review did not effectively utilize key features of learning. This report outlines our innovative approach to rejuvenating board review utilizing principles of adult learning, self-motivation, and game-based learning.

Within the realm of game-based learning, "gamification" involves the application of game design elements (conceptual building blocks integral to building successful games) to traditionally nongame contexts [1]. Friedlander et al., in 2011, described direct links between the neurobiology of learning and medical education [2]. They articulate concepts such as repetition, reward, reinforcement, active engagement, and stress, all of which can be seen during a game-based activity [1,2]. With respect to the learning environment, Rutledge et al., in 2018, described gamification as a practical consideration for medical educators [1]. They qualified the concept of gamification with respect to medical education as a valuable learning tool, facilitating and motivating the learner’s progression to self-determination and intrinsic motivation [1].

Given the basis for gamification as a useful learning technique, the value and perception of game-based learning have been described with an emphasis on engagement, learning climate, and learner perspectives [3-11]. Studies have shown modest improvements in knowledge and surgical skills with respect to gamified educational systems; most of this work has been done in subspecialties within Internal Medicine and Surgery [12-26]. These studies use primarily electronic platforms and do not commonly employ real-time directly competitive games, other than escape rooms. To date, there are no studies on the benefits and effectiveness of game-based learning within Internal Medicine residency programs pertaining specifically to resident board review and longitudinal didactics.

With these concepts in mind, we designed, implemented, and evaluated a longitudinal, team-based, game-based board review competition, the “Cohort Cup”. The “Cohort Cup” was intended to facilitate a learning environment rich in the aforementioned principles of medical education. We hypothesized that our “Cohort Cup” would improve resident attendance at board review sessions, resident perceptions of the value of board review, and motivation to prepare and attend.

## Materials and methods

A junior faculty member with a passion for games developed the “Cohort Cup”. He proposed an outline of the longitudinal competition to the residency leadership and which was subsequently approved by the residency operations committee. The faculty champion worked closely with chief residents and used the American College of Physicians Internal Medicine in-training exam (ITE) score report to identify key topics that traditionally had been problematic for residents. The game-based intervention occurred weekly from November 2017 through May 2018, excluding holiday weeks. It was held in the central academic conference room for one hour during the weekly academic half-day educational didactics. Our residency program comprised 105 eligible (categorical Internal Medicine) residents and assigned residents to one of five cohorts for scheduling purposes; this preassigned structure of cohorts formed the basis of our team-based “cohort” competition. Each team comprised an equal number of PGY-1, PGY-2, and PGY-3 residents.

To begin, each cohort received supplies to create a coat-of-arms poster to symbolize their cohort’s sense of comradery within the residency; these were prominently displayed in the conference room throughout the competition. Inspired by popular game shows and childhood games, we adapted classic games that facilitated group learning. A variety of games were used, including a Jeopardy-like game, celebrity password, virtual scavenger hunt, and others (see Appendix 2 for templates and rules for each game). Each game-based session lasted for one hour, and all games utilized high yield board-review information from the American College of Physicians' Medical Knowledge Self-Assessment Program (MKSAP). The faculty champion was present for each learning game with a referee’s jersey, a whistle, and a stopwatch to facilitate control over the learning environment. Penalties were assigned and flags tossed into the gaming field whenever an “unsafe learning” event or situation presented itself (e.g., residents verbally berating a fellow teammate for losing a point). This ensured that a supportive, competitive spirit of gamesmanship was maintained and protected against a potentially unsafe learning environment. At the end of the competition, a “Cohort Cup” trophy was presented to the winning cohort, and they were taken out to dinner by program leadership, including the Program Director, Associate Program Directors, and the Vice-Chair for Education.

We used two leaderboards (visual representations of team scores) throughout the Cohort Cup. The first leaderboard consisted of five glass vases lined up in the conference room where the Cohort Cup was held. Each team’s vase held colored beads corresponding to their cohort’s color that reflected the number of total points they had accumulated thus far in the competition. Additionally, the hand-crafted Cohort Cup trophy itself served as a leaderboard. It featured a multicolor LED light that would shine the color of the cohort that was currently in the lead and was stationed in the central conference room (where most residents would see it daily).

Pre- and post-surveys were developed by physician educators with experience in survey design, modified iteratively, and piloted for usability. Surveys measured resident attitudes regarding self-perceptions of engagement, quality, value, and senses of peer bonding. Our survey was created internally to directly measure the above attitudes. (see Appendix 1 for pre/post surveys). The surveys utilized a 5-point Likert scale: 1 = strongly disagree, 2= disagree, 3= neutral, 4=agree, 5= strongly agree. Analysis comparing mean pre/post response value was conducted, and our results were statistically significant with 95% confidence and p-value ≤.01 for all questions reported. The surveys also contained a field for “Additional Comments” where participants would provide their own thoughts and opinions regarding the intervention.

The Wayne State University IRB granted us an educational innovation exemption. Residents were made fully aware of the optional nature to participate.

## Results

Throughout the seven-month intervention period, 22 “Cohort Cup” sessions occurred, including a three-hour grand finale. 

Of 105 eligible residents, 82 completed the pre-intervention survey, and 74 completed the post-intervention survey (78% and 70% response rate, respectively). Results are shown in Table 1. We found statistically significant improvements in resident self-perceptions of engagement during board review, perceived educational value of board review, and preferences for game-based learning in board review after the intervention. Self-perceptions of learner engagement improved from 2.74 to 3.8. (p=1.307e-8). The value of the educational experience increased from 2.68 to 3.95. (p=1.97e-12). Preferences for game-based learning improved from 3.77 to 4.32. (p=5.4e-4). These data represent resident averages pre- and post-intervention.

**Table 1:** Cohort Cup Pre/Post Survey Results Pre-intervention and post-intervention survey results.  As denoted, 82 pre-surveys collected and 74 post-surveys were collected.  The number of residents eligible to take the survey = 105. (78% pre-intervention response rate, and 70% post-intervention response rate).  Surveys were collected electronically with no incentive for voluntary participation.  Analysis was conducted via t-test with 95% significance. (Last four survey items were not assessed on the pre-intervention survey) 1 - Strongly Disagree, 2 - Disagree, 3 - Neither Disagree nor Agree, 4 - Agree, 5 - Strongly Agree N/A=Not applicable.

Survey Item	Pre-intervention Average (SD) (N = 82)	Post-intervention Average (SD) (N=74)	P-Value
I am engaged in the academic half-day as they are currently designed.	2.74 (1.195)	3.8 (1.007)	P = 1.307e-8
The current structure of the academic half-day contributes to my education.	2.68 (1.121)	3.95 (.949)	P = 1.97e-12
I prefer games and active learning as opposed to standard lecture-based didactics for my learning.	3.77 (1.087)	4.32 (.853)	P = 5.4 e-4
I prefer the “Cohort Cup” activity as opposed to standard lecture-based didactics for my learning.	-	4.24 (.957)	N/A
I feel that I learn more from the “Cohort Cup” activity than I do from standard lecture-based didactics.	-	4.03 (.986)	N/A
I prefer to have the “Cohort Cup” remain part of my didactics.	-	4.42 (.725)	N/A
I feel that the “Cohort Cup” activity has helped me bond with others in my residency	-	4.36 (.769)	N/A

Additionally, we investigated post-intervention preferences for the cohort-cup to remain as a core component of the resident board-review curriculum and also investigated the effect of the cohort-cup on resident bonding. We observed an average score of 4.42 for preferences for the “Cohort Cup” to remain present in resident board review and an average score of 4.36 regarding positive effects on resident bonding. Average attendance doubled from 12 residents per session during the previous traditional didactic board review to 25 residents per session for the “Cohort Cup” session. We also received comments on the survey reflecting residents’ increased enjoyment, engagement, and sense of comradery. These perceptions were also articulated via informal meetings regarding feedback after the intervention.

One faculty was present for each game, and, during our intervention, we observed that residents began asking the faculty host for topics ahead of time so that they could prepare for the sessions. It was noted that one resident came in on their day off to participate and help their team win that day. Our board passage rate increased from 86% the year before the “Cohort Cup” to 97% the year of the “Cohort Cup”.

## Discussion

The response to the “Cohort Cup” was promising; our game-based, team-based, longitudinal intervention yielded improvement in perceptions of multiple metrics, including perceived effectiveness, engagement, and value of learning games. We also improved the board review attendance. The faculty noted that the gamified sessions resulted in numerous positive interactions amongst and between the cohort members. Gamified systems have the potential to significantly positively impact learning climate, learner engagement, and relatedness [1-11]. We believe the "Cohort Cup" accomplished this mission by appropriately facilitating a competitive environment to harness the potential of game-based learning.

Aside from the quantitative survey data presented above, we observed palpable changes in energy regarding the gamified board review. The "Cohort Cup" became a regular topic of conversation within our department and served as a vehicle to untie residents and facilitate teamwork and comradery. People identified by their "Cohort Cup" color and even came to morning reports dressed in their team colors! They developed cheers that were heard while waiting for conferences to begin. Literature supports that game-based learning positively impacts team building and comradery, and we noticed this with our program [1,2,6-7,9]. We observed that residents would attend in higher numbers, even when they were on busy inpatient services. Residents were fully invested in helping their cohort win, and were doing so with positive energy and outward demonstrations of joy during the games. Additionally, residents demonstrated on the survey that the activity improved their sense of bonding, a finding likely related to the intrinsic interactive game-based and team-based design and indicative of the principle of relatedness concerning the value of the learning environment [1,2]. The “Cohort Cup” brought an educational zest back to the educational paradigm within the program. With respect to competition, it also demonstrated the effective use of safe competition to facilitate engagement and motivation. This analysis shows that such a gamified educational intervention, rooted in real-time direct competition, can contribute positively to the educational culture within a residency program.

Several elements contributed to the success of the “Cohort Cup”. First, a faculty champion was essential. Analogous to a board review director, the champion identified relevant board review material, created games, and held a constant presence during each session. Additionally, the champion facilitated a lively, competitive atmosphere while preserving the integrity and safety of the learning environment for all learners. Second, the use of leaderboards both during and in-between “Cohort Cup” sessions contributed to a sense of competition that was essential to the success of the “Cohort Cup”. Their pervasiveness throughout the year also contributed to the motivation and structural permanence of the “Cohort Cup”. Finally, program leadership was fully supportive of the project and willing to experiment with a new paradigm. 

There are some limitations to our intervention and analysis. Firstly, we present the results of a single program. We do not know if our residents are more game-oriented than other residents at other programs. Second, there may have been some selection bias. Residents who are competitive and game-oriented may not attend a traditional board review and be more likely to participate in a competitive board review. Finally, the champion was vested in the success of the project. Any board-review structure led by a committed and engaging facilitator would result in a more engaging and satisfying board review, even without having a gamified basis. Another major determining factor, and possible limitation, is that the intervention is only as strong as the residents’ willingness to participate, given the key principle of learner autonomy as an integral component of autonomy within the concept of self-determination [1]. As such, the voluntary nature of the “Cohort Cup” and the spirit of the moderator/environment becomes paramount to garnering participation.

Given the success of the “Cohort Cup”, we have set out to further quantify and qualify the effects of gamified learning environments. Currently, we are amidst analysis of a focus group-based formal qualitative analysis of a similar implementation of gamified learning. We are assessing a longitudinal game-based board review curriculum at a second institution, the University of Pittsburgh in the Department of Pediatrics, to further assess the learning environment of such an innovative curricular paradigm. Additionally, we have connected with a few institutions nationwide to continue to build a platform for game-based education within medical education. We hope that the attached templates serve as a basis for programs to engage in such a worthwhile endeavor. Given the recent shift to virtual education, as many programs begin to resume in-person didactics, we hope that programs will choose to incorporate innovative game-based sessions within their curricular paradigms.

## Conclusions

Our longitudinal team-based, game-based board review paradigm, the “Cohort Cup”, successfully improved resident perceptions of board review quality and also improved resident attendance. Board review has become an engaging environment that residents seem to enjoy more. Game-based learning has become a mainstay for our board-review curriculum delivery and could potentially be adopted by other residency programs.

## Appendices

Appendix 1

Pre- and Post-Surveys

Cohort cup pre-survey:

1.       I am engaged in the academic half-day as they are currently designed. Strongly Disagree            Disagree              Neutral               Agree                  Strongly Agree

2.       Faculty are engaged during the academic half-day as they are currently designed. Strongly Disagree            Disagree              Neutral               Agree                  Strongly Agree

3.       I look forward to the academic half-day. Strongly Disagree            Disagree              Neutral               Agree                  Strongly Agree

4.       The current structure of the academic half-day contributes to my education. Strongly Disagree            Disagree              Neutral               Agree                  Strongly Agree

5.       I prefer games and active learning as opposed to lectures for my learning. Strongly Disagree            Disagree              Neutral               Agree                  Strongly Agree

6.       Having a “game” component to the academic half-day would be beneficial to my learning. Strongly Disagree            Disagree              Neutral               Agree                  Strongly Agree

7.       I prefer case-based didactics. Strongly Disagree            Disagree              Neutral               Agree                  Strongly Agree

8.       I prefer team-based learning didactics. Strongly Disagree            Disagree              Neutral               Agree                  Strongly Agree

9.       I have experienced a “flipped classroom” method of teaching. Strongly Disagree            Disagree              Neutral               Agree                  Strongly Agree

10.   Feel free to give any suggestions for the cohort cup/resident education.

Cohort cup post-survey:

1.       I am engaged in the academic half-day as they are currently designed. Strongly Disagree            Disagree              Neutral               Agree                  Strongly Agree

2.       Faculty are engaged during the academic half-day. Strongly Disagree            Disagree              Neutral               Agree                  Strongly Agree

3.       I look forward to the academic half-day. Strongly Disagree            Disagree              Neutral               Agree                  Strongly Agree

4.       The current structure of the academic half-day, with the addition of the cohort cup, contributes to my education. Strongly Disagree            Disagree              Neutral               Agree                  Strongly Agree

5.       I prefer games and active learning as opposed to lectures for my learning. Strongly Disagree            Disagree              Neutral               Agree                  Strongly Agree

6.       I prefer the cohort cup activity as opposed to standard lecture-based didactics for my learning. Strongly Disagree            Disagree              Neutral               Agree                  Strongly Agree

7.       I feel that I learn more from the cohort cup activity than I do from standard lecture-based didactics. Strongly Disagree            Disagree              Neutral               Agree                  Strongly Agree

8.       Having a “game” component to the academic half-day, i.e., the cohort cup, is beneficial to my learning. Strongly Disagree            Disagree              Neutral               Agree                  Strongly Agree

9.       I prefer to have the cohort cup remain part of my didactics. Strongly Disagree            Disagree              Neutral               Agree                  Strongly Agree

10.   I feel that the cohort cup activity has helped me bond with others in my residency. Strongly Disagree            Disagree              Neutral               Agree                  Strongly Agree

11.   I feel a greater sense of collegiality and teamwork having participated in the cohort cup than I felt prior to participation with the cohort cup. Strongly Disagree            Disagree              Neutral               Agree                  Strongly Agree

12.   Please provide any additional feedback on the cohort cup experience.

13.   Feel free to give any suggestions for the cohort cup/resident education.

Appendix 2

Game Templates

The following game templates were used:

1.   Medical Jeopardy-Like Game 2.  Celebrity Password 3.  Celebrity Hollywood Squares 4.  Sarah’s Cups 5.  Virtual Photo Scavenger Hunt

1.  Medical Jeopardy-like game

Overview: Teams take turns to select categories and point values to answer questions. Best in teams of 4-6; can accommodate any number of teams. End Condition:  After one or two rounds of Jeopardy followed by a final Jeopardy (wager-based) question, the team with the highest number of points wins.

Supplies: Jeopardy Template: Free Jeopardy PowerPoint template available here: https://face2faceyouthgroup.com/events/jeopardy-game/ (Author of the game permits for public use).  Per Team: Mini dry erase board, dry eraser, dry erase marker.

Gameplay: The gameplay is conducted with a standard Jeopardy board.  In our experience, five categories of five questions each work best.  Point values increase, as does difficulty: 100, 200, 300, 400, and 500.  If a second round is used, the point values are 200, 400, 600, 800, 1000.  Teams take turns being the action team.

The first action team chooses a category and point value (e.g., “Liver Disease for 500”). The question is read.  All teams have the 30s to 1 min to write down the answer on their boards (you can choose whatever time limit works best for you).  After the allotted time, the action team (the team who chose the category) decides how they want to proceed.  They can attempt to answer, or they can pass (not attempt to answer):

If they answer correctly, they are awarded the point value of that question, the concept is reviewed as necessary, and it is the next action team’s turn to choose a category.  If they answer incorrectly, they lose the points that they attempted and every other team with the correct answer written on their board receives 100 points.  If they choose to pass, they neither gain nor lose points, and all teams with the correct answer written on their boards receive 100 points.

Notes: Teams who attempt to earn their 100 points when the action team either passes or gets it wrong does not lose points if they are wrong.  They essentially get a free guess at 100 points. Boards must be held by the player on the team of the lowest training level - if the host asks that person (or any member of the team) to explain an answer at any time, and the person asked CANNOT explain the answer, then points get revoked. This emphasizes that the point of the game is to reinforce the tested concepts/questions and motivates teams to work together to ensure that all members understand the material.  This also helps provide a framework for senior-level residents to assume a managerial role and educator role.

Facilitator Tips: Always ask each team what their answer is to every question.  Even if team A has chosen the category/point value and chooses to answer the question, it is good practice to ask all other teams to show their boards and acknowledge all answers before revealing the correct answer. This allows the facilitator to provide the most relevant context/explanation of the answer and also works to keep all teams actively engaged. Use phrases such as “I’m hearing your team’s answer as…”, “Thank you for your answer…”.  It is detrimental to the learning environment to use phrases such as “That answer is correct… (said before eliciting the other teams’ answers)” or “Why would you say that?” Ensure a zero-tolerance policy for bullying and unsafe learning environment (putting opponents down, teams calling each other names).  Recommend the use of “penalty flags” like a football game where the facilitator can throw them out and enforce an “unsafe learning” penalty.

2.  Celebrity password (played like the classic TV gameshow password)

Overview: Teams attempt to guess a mystery word (the “password”) and are subsequently given a five-question mini quiz related to the “password”. Best in groups of 4-6, can accommodate as many teams as necessary.  End condition based on rounds: usually 4 to 6 rounds take about one hour. 

Supplies: Each Team Needs: Hand-held dry erase board. Dry-erase marker. Dry eraser. Host Needs: Predetermined “passwords”. Celebrity clue giver(s) are allowed to know the passwords ahead of time to prepare or can be informed in real-time. Mini-Quizzes: Each password has a corresponding five questions mini quiz that gets administered to each team after a password is successfully identified.

Game Play: Each team is paired up with a “celebrity” (a faculty member, fellow, etc.). If there are not many “celebrities” available, one “celebrity” can give a password that is played by every team simultaneously.

All celebrities are shown a password (e.g., Lactulose). The first celebrity says one clue (it is only allowed to be one word) out loud for everyone to hear (all teams can hear).  The first celebrity’s team, and only that team, get a chance to identify the password by writing a word on their dry-erase board.

If they are incorrect, then the next celebrity states a second word out loud for all to hear, and that celebrity’s team then has a turn to guess the password. Play continues like this until a team correctly identifies the password. Once a team correctly identifies the password that team receives two points, and the password is identified. The mini-quizzes are then handed out to each team (or it can be displayed on a monitor), and teams have five minutes to answer the five-question mini-quiz.  Teams work together (in their respective teams). Of note, the quiz is designed around the password.  So, in this example, the quiz would be about liver disease, AASLD guidelines, cirrhosis, hyperammonaemia, etc.

After five minutes, the quiz is reviewed orally by the host with answers and explanations.  Teams receive one point for each question they answered correctly (keeping in mind that the team who identified the password received two extra points). After the mini quiz has been explained and points for that round are awarded, a new round begins.  Of note, the order in which celebrity/team pair goes first simply rotates from team to team.  It is of no importance which team guessed the word right.  For round one, the first celebrity team/pair goes first.  For round two, the second pair goes first.  It does not matter which team guessed the word correctly during the prior round. If there is only one celebrity available:

The celebrity gives a clue, and each team writes a guess at the password on their whiteboards.  Host checks them all without making it known which team has the correct password if any. The celebrity then gives a second clue, and the host again checks all boards. The celebrity then gives a third clue, and the host then checks all boards for a final time.  If a team guessed the password correctly after only one clue, they get three points.  After two clues, they get two points.  After three clues, one point.

Facilitator Tips: Circulate throughout the room while repeating the clue, i.e., if the celebrity gives a clue as “jaundice” for a password of “bilirubin”, then repeat the clue “jaundice” as you circulate the room to ensure that all teams have heard the password correctly. When only one celebrity is present: It is OK to give a discreet nod if a team has appropriately identified a password as you circulate the room.  Avoid openly expressing when a team has identified the correct password as to not distract other teams who are actively trying to still figure out the password.

Once all three clues have been given, and all teams have taken their third guess (if they haven’t already identified the password), then clearly and explicitly identify what the password was, so teams are very clear on what they are about to be quizzed. Hand out the quizzes face down and then announce a starting time.  Hand the quizzes directly to the member of the team of the lowest training level.

When reviewing answers to the quiz (question by question), ask every team for their answer before going over the correct answer (without identifying their answers as correct or incorrect when they report them). This helps normalize the behavior of answering without the pressure of correctness in front of the group. Ensure a zero-tolerance policy for bullying and an unsafe learning environment (putting opponents down, teams calling each other names). Recommend the use of “penalty flags” like a football game where the facilitator can throw them out and enforce an “unsafe learning” penalty.

3.  Celebrity Hollywood squares (played like the classic TV gameshow Hollywood squares)

Overview: Any number of teams: For each round: two “action” teams play head-to-head. All other teams (If there are more than two) are audience members. Audience teams also answer questions that are posed to the “action” teams and can earn points for their teams even while they are not the “action” team. Teams pick a square on a tic-tac-toe board that is marked with a faculty name.  That faculty member then answers a question (can tell the truth or deliberately lie), and the action team must “agree” or “disagree” correctly, with that faculty member’s answer to earn that square. The first team to achieve tic-tac-toe wins the round.  (If no tic-tac-toe develops, then the first team to win five total squares wins via a “five-square-win”.) Best in teams of 4 to 6 can accommodate two teams playing head-to-head, but other teams can still participate as audience members. End condition based on rounds: usually 2 to 3 rounds comprise about one hour.

Supplies: Tic-tac-toe board displayed largely in front of everyone (e.g., ppt, or drawn on a board). A physical token that can be affixed to the tic-tac-toe board - one token for each team. One team can be Xs, and the other team can be Os. Predetermined questions that the celebrity is usually aware of well in advance:

The questions are intricate, more so than for other games: i.e., “According to the latest ACC/AHA guidelines on heart failure, please explain the change in the recommendation on the utility of Spironolactone in heart failure with reduced ejection fraction (HFrEF)”. ALL TEAMS who are watching/waiting for their turn need a dry-erase board.

Gameplay: The two teams take turns. The action team picks a square on the board to attempt to earn that square:

If there are multiple celebrities, then the celebrity’s names are written in the square ahead of time, so the team knows with which faculty/fellow they are playing to earn that square. Teams MUST announce if they are attempting a square for the Win, five-square win, or block (just like the classic TV show). “I’d like Dr. Lee’s square for the win”, for example. Once the team picks a square, a question is read out loud (see above for an example). These are usually specific evidence-based questions that require critical reasoning/analysis to answer. They can also be case-based as long as the question has an unequivocal answer. After the team discusses for 30 seconds to a minute (and all other teams are also discussing), the celebrity gets to state an answer: Example “well host, I recently read those guidelines, and I was surprised to learn that Spironolactone is now proven to be mortality reducing for many more patients, and, as such, should be given to all patients with HFrEF who have NYHA Class II-IV heart failure!”

The answering celebrity should be deliberately misleading and tricky! The action team then takes the 30s or 1 min (whatever works best for your environment) to discuss the celebrity’s answer and subsequently must either “agree” or “disagree” with the celebrity.  If they choose correctly and can explain why then they earn their square and that square gets marked with their token as belonging to that team.

The first team to tic-tac-toe, or a five-square win, wins the game and 50 bonus points. While the action team is deliberating whether or not to agree or disagree, each team in the audience should also be deciding if they agree or disagree with the celebrity.  After the host asks the action team for their decision, all teams in the audience should be prompted to raise their boards and show their decisions (agree or disagree).  Each correct decision/response by every team who isn’t on the action team earns 10 points (that includes the opponent of the action team for that round).

Facilitator Tips: Be sure to have every team who isn’t on the action team write “agree” or “disagree” on a whiteboard during gameplay for each question. Review the correct answer out loud. This can be done by the host, or the host can ask for a volunteer to state their team’s explanation. Ensure a zero-tolerance policy for bullying and an unsafe learning environment (putting opponents down, teams calling each other names).  Recommend the use of “penalty flags” like a football game where the facilitator can throw them out and enforce an “unsafe learning” penalty.

4.  Sara’s cups

Overview: Any number of teams: Works best in large teams (5-10). Teams send a “tribute” to the center where there are different category cups filled with slips of paper that each has one question written on them. Tributes get to pick a question from whichever cup they like, and their team must answer - other teams must attempt to answer if the action team gets it wrong! End condition based on rounds: completing 10-15 questions takes about one hour. 

Supplies: Three cups (categories) filled with various questions about that category written on small slips of paper in the cups (each cup’s category is displayed outside the cup, so everyone knows which cup corresponds to which category of questions). Cup A = “primary care screening guidelines” and is filled with 15 small slips of paper, each with a question re: primary care screening guidelines on them. These cups need to be placed on a table that is easily accessible and, in the center, or in front of all the teams (and not too close to anyone's team). Each team: Mini dry erase board. Dry erase marker. Dry eraser.

Gameplay: Teams take turns going first. Team A’s turn: Team A selects one “tribute” to approach the cups and draw a question from whatever cup they like.  They read the question out loud to everyone in the room.

After 30 seconds, the tribute from Team A, who is still standing alone at the cups, can choose to try to answer the question independently or can choose to consult with their team (if they choose to consult, they get a 30-second consultation and then must answer the question).  They must stay next to the cup this entire time. If they answer correctly alone, their team is awarded five points, and it’s the next team’s turn. If they answer correctly with a consultation, their team is awarded two points, and it’s the next team's turn. Of note, the tribute only gets one guess, period. If they attempt to earn five points by answering solo but get it wrong, their turn is over.  They cannot then consult their team for a second guess. If they answer incorrectly: No loss of points for the team who picked the question. This starts the chain reaction: Having answered incorrectly, the tribute gets to choose a different team, who then must attempt to answer the question (if team A’s tribute gets it wrong, then team A’s tribute must call on any other team, team D for example, and then team D, is then forced to answer the question).

If that team (team D) is wrong, they get negative two points and they get to call on a subsequent team.

That subsequent team must then answer.  If they are wrong, they lose two points, and they call on any not-yet chosen team. This pattern continues until either: A) a team gets it correct (and is awarded two points).

B) all teams get it wrong (meaning, every team, except the team who started it [team A in our example], lost two points). After the question’s answer is resolved by either a team answering correctly or all teams answering incorrectly and the host explaining the answer, the next team goes. Gameplay is in sequential team order.  So, despite which team earns the points on team A’s turn, team B will always start the next round with their tribute. And then team C will start the next round, and then D, and so on.

Facilitator Tips: Help to acknowledge and provide supportive comments to tributes as they approach the selection bin. High-fives and positive gestures (thumbs-up) can help reflect elements of fun and support.  It can be a little nerve-wracking to have to pick a question in front of the whole group. Re-read the question after the tribute reads the question allowed and clearly announce the timing and the tribute’s decision to attempt to answer or consult with their team. Verbalize all decisions by the tribute so that every participant of every team can hear what is happening. Identify the answer as correct or incorrect directly, with no commentary, when the answering tribute/team answers it.  Move on to the next team (in the event of an incorrect answer) fluidly with minimal emphasis on the fact that the answer was wrong.

Ensure a zero-tolerance policy for bullying and an unsafe learning environment (putting opponents down, teams calling each other names). Recommend the use of “penalty flags” like a football game where the facilitator can throw them out and enforce an “unsafe learning” penalty.

5.  Virtual photo scavenger hunt (high energy and very fun!)

Overview: Any number of teams: 4 to 6 member teams, maximum teams receive a scavenger hunt list of evidence-based questions (all teams receive the same list). Each question is paired with instructions for a selfie (i.e., “take this picture with someone who works in admitting”). Teams race around the hospital with a dry-erase board and their lists.  For each item on their list, they must write the correct answer on their dry erase board and then text a selfie to the host in the manner described by the question (i.e., for the above example, a team would need to write down the correct answer to that question on their dry-erase board and then text a selfie to the host of their team holding the correct answer written on their board with someone from admitting). Every time a team successfully texts a selfie, the host responds to that team with a letter.  All of the letters unscramble a location where the team must reach to win!

Team-based, best in groups of 4-6. End condition (whichever comes first): a team reaches the final location and wins the game. A predetermined time limit is reached, and the hunt ends - the team with the most correct selfies wins.

Supplies: Each team needs a portable legal-size dry erase board and marker. One team member on each team needs a device that can message the game host. Each team needs a scavenger hunt list.

Gameplay: Prior to the start of the scavenger hunt: each team elects a messenger whose job it is to text message the game host. Each team receives a whiteboard and marker. Each team receives one scavenger hunt list. The scavenger hunt list contains medically relevant questions. Each question is paired with instructions/criteria for taking a selfie. [Example:  Name two criteria that can indicate someone’s C. Diff as fulminant. Take this photo with the nurse for any of your patients]. During the scavenger hunt, while the teams are busy taking their photos, the game show host should remain in the room where the hunt starts with a computer and the ability to rapidly respond to each team’s chosen messenger.

Once the scavenger hunt starts, teams have to come up with the answers to the questions (they are allowed to use primary literature to help them [and use their devices on the go]).  When they think they know the answer to one of the questions, they have to write it down (they can answer the questions in any order they wish) on their whiteboard and take and send (to the host) a team selfie in the manner that matches the instructions given with that question: [in the above example:  when the team thinks they have discovered the answer to that question, they must write their answer down on their whiteboard and send a team selfie to the game show host with any one of their nurses (the whiteboard with the correct answer must be visible in the selfie). If the answer is incorrect, the game show host must respond immediately with “incorrect”. If the answer is correct, the game show host responds with a single letter: “t” for example. Every time the team messenger messages a correct selfie/answer combination, the game show host sends a different letter (each question can only be correctly answered one time by each team). As teams collect letters, they are trying to unscramble the location of the final challenge.  [i.e., letters can spell c-a-f-e-t-e-r-i-a or l-i-b-r-a-r-y] Once a team has enough letters to think they know where the final challenge is located, they can go there.  Someone should be waiting at the final challenge location with a final medical knowledge question (usually, this is more involved, like a full case scenario or multi-step question). When the team arrives at the final challenge location, they are afforded one chance to answer the question (they are not allowed devices nor primary literature to help them).  (A co-host is usually waiting at the final challenge location to administer the final challenge.  The main host of the scavenger hunt is usually in the room where the educational session starts/ends).

If the team gets the final challenge correct, the scavenger hunt is over, and the game show host should message the messenger of every team to announce that a team has won so all participants can return to the room. If the team gets it incorrect, they are not allowed to reattempt until they complete their scavenger hunt form (if it is already completed, then they have to wait until at least one other team attempts the final challenge and fails). After their second try, they cannot attempt again until two teams try and fail. Once teams return to the room (after one team answers correctly or when the time expires, whichever happens first), then all answers are discussed with emphasis on primary literature.

Facilitator Tips: Make sure the internet connection is of high quality and that messaging service is highly functional within your location. Keep an open and active line of communication with the final challenge co-host.

This is a fast-paced activity that requires complete concentration on behalf of the facilitator. It is very helpful to have a co-facilitator/helper (observing chat windows, keeping track of where the final challenge is located and which team(s) have approached/tried to answer, helping corral teams back to the educational room when the hunt is over). After all, teams have returned to the educational room, acknowledge the winning team, but place more emphasis on group review of the material.  Be sure to ask a few teams for their answers to each question to facilitate a culture of answering and inquiry.

Emphasize literature and where answers are coming from. Ensure a zero-tolerance policy for bullying and unsafe learning environment (putting opponents down, teams calling each other names).  Recommend the use of “penalty flags” like a football game where the facilitator can throw them out and enforce an “unsafe learning” penalty.
